# GPD1L downregulation in colorectal cancer: a novel obesity-related biomarker linking metabolic dysregulation to tumor progression

**DOI:** 10.3389/fonc.2025.1582728

**Published:** 2025-06-05

**Authors:** Feng Zhu, Huiyuan Li, Hongzhang Liu, Yusheng Wang

**Affiliations:** ^1^ General Surgery Department of Jincheng People’s Hospital, Shanxi, China; ^2^ General Surgery Department of Jincheng Hospital Affiliated to Changzhi Medical College, Shanxi, China

**Keywords:** metabolism-associated gene, colorectal cancer, GPD1L, bioinformatics, tumor suppressor genes

## Abstract

**Objective:**

To delineate the expression profile and tumor-suppressive function of the metabolism-associated gene GPD1L in colorectal carcinogenesis. Methods: Transcriptomic datasets from TCGA and GEO repositories (GSE74602, GSE113513, GSE164191) were computationally analyzed. Paired tumor/adjacent mucosal specimens (n=58) from CRC patients at Jincheng People’s Hospital were analyzed alongside the NCM460 colon epithelial line and five CRC lines (SW620, HCT116, SW480, DLD-1, LOVO). Following GPD1L quantification via qPCR, selected cell models underwent pcDNA3.1-GPD1L transfection for functional characterization. Then Western blot analysis was used to explore its possible mechanism.

**Results:**

Comparative analysis revealed a marked elevation of GPD1L expression in non-neoplastic tissues relative to tumor specimens (P<0.001). Transcriptional profiling further identified significant depletion of GPD1L mRNA levels across malignant cell lines versus the NCM460 epithelial reference (P<0.05), with HCT116/SW620 showing maximal downregulation. Ectopic GPD1L expression attenuated oncogenic phenotypes: proliferation decreased (P<0.001), while Transwell quantification revealed 46.0% (HCT116: 605.0 ± 9.2 vs 326.7 ± 8.50 cells/field) and 54.3% (SW620: 455.3 ± 17.2 vs 208.0 ± 14.0 cells/field) reductions in migratory capacity (both P<0.001). Invasion assays showed parallel inhibition (HCT116: 43.3% decrease, P<0.01; SW620: 54.8% decrease, P<0.001). After overexpression of GPD1L, the expression levels of HIF-1α and MMP9 were reduced (P<0.05).

**Conclusion:**

GPD1L downregulation represents a hallmark of CRC progression, with affecting the expression of HIF-1α and MMP9 significantly impeding malignant behaviors, nominating it as a candidate tumor suppressor in colorectal neoplasia.

## 1 Introduction

Globally, colorectal adenocarcinoma ranks as the second most prevalent malignancy, with epidemiological surveillance data from the 2022 GLOBOCAN initiative documenting approximately 1.9 million incident cases and 935,000 mortality events annually. This disease burden translates to nearly 10.6% of total cancer-related deaths worldwide, establishing it as a critical public health priority in oncological research (1). The disease pathogenesis involves complex interactions between hereditary predisposition and modifiable lifestyle factors. Germline cancer predisposition syndromes, predominantly characterized by Lynch syndrome and familial adenomatous polyposis, contribute to approximately 5-10% of CRC pathogenesis. These high-penetrance autosomal dominant disorders collectively represent a significant subset of hereditary CRC etiology (2). While lifestyle determinants including sedentary behavior, excess adiposity, and ethanol consumption demonstrate dose-dependent associations with CRC risk (3, 4). Of particular concern, epidemiological meta-analyses establish obesity as an independent risk modulator for CRC development (OR=1.33, 95%CI:1.21-1.46) (5–7), but the precise pathophysiological pathways linking excess adiposity to colorectal oncogenesis have yet to be fully elucidated.

Originally identified in 2007 through its modulation of voltage-gated sodium channel (Nav1.5) membrane localization in cardiomyocytes, the glycerol-3-phosphate dehydrogenase 1-like (GPD1L) has evolved from its canonical characterization to be recognized as a pleiotropic metabolic orchestrator with multifunctional regulatory capacities (8). Beyond its established role in Brugada syndrome pathogenesis through SCN5A modulation (9), recent investigations reveal GPD1L’s capacity to destabilize the oxygen-sensitive transcriptional regulator HIF-1α via prolyl hydroxylase (PHD) activation (10). Notably, adipose tissue GPD1L expression exhibits dynamic regulation during nutritional interventions - upregulated 2.1-fold during calorie restriction (P<0.01) versus suppressed 63% under high-fat feeding (P<0.001) (11). This highlights GPD1L’s candidacy as a master metabolic checkpoint, offering novel opportunities for developing small-molecule therapies against lipidomic disorders and glucoregulatory dysfunction.

This regulatory duality positions GPD1L as a compelling molecular interface between energy homeostasis and oncogenesis. Building upon these observations, we hypothesize that GPD1L dysregulation may mechanistically connect obesity-associated metabolic dysfunction with CRC progression. Our investigation systematically evaluates: (i) the differential expression patterns of GPD1L in CRC versus normal mucosa, (ii) its clinicopathological correlations, (iii) functional consequences of GPD1L reconstitution on malignant phenotypes *in vitro*, and (iv) the molecular mechanism of GPD1L influencing CRC cells.

## 2 Materials and methods

### 2.1 Transcriptomic profiling and clinical data curation

RNA sequencing datasets formatted in HTSeq-FPKM units, encompassing colorectal adenocarcinoma (COAD), rectosigmoid junction carcinomas and rectal adenocarcinoma (READ), were systematically curated from The Cancer Genome Atlas (TCGA) database (retrieval date: December 3, 2022). The primary cohort comprised 554 neoplastic samples juxtaposed with 48 histologically normal controls. To ensure analytical rigor, specimens with incomplete clinicopathological metadata were excluded through a standardized filtration protocol. Post-curation cohorts were stratified into two analytical subsets: 533 cases allocated for survival trajectory modeling and 467 cases subjected to Cox proportional hazards regression. For external validation, three orthogonal transcriptomic repositories (GSE74602, GSE113513, GSE164191) were acquired from the Gene Expression Omnibus (GEO) platform.

### 2.2 Clinical specimen collection

Fifty-eight treatment-naïve CRC patients undergoing curative resection at Jincheng People’s Hospital (January 2023-January 2024) were prospectively enrolled. Surgically resected tumor specimens paired with histologically normal adjacent tissues (harvested from macroscopically normal regions ≥5 cm distal to the neoplastic periphery) were subjected to rapid cryopreservation via liquid nitrogen immersion within 10 minutes of resection. Processed biospecimens were subsequently transferred to vapor-phase liquid nitrogen cryogenic storage systems maintained at -80°C. Inclusion Parameters: (1) histologically confirmed adenocarcinoma; (2) R0 resection; (3) AJCC 8th edition staging available. Exclusion Parameters: neoadjuvant therapy history. All patients’ specimens were collected and archived under protocols received formal ethical certification (IRB Approval No.: JCPH-20240724007) from the Jincheng People’s Hospital.

### 2.3 RNA quantification

Tissue samples were homogenized in TRIzol^®^ reagent (Invitrogen) followed by RNA extraction using chloroform-isopropanol precipitation. cDNA synthesis was performed with HiScript III RT SuperMix (Vazyme Biotech). qPCR amplification employed ChamQ SYBR Master Mix (Vazyme Biotech) under standardized conditions: the amplification regimen was initiated by a 60-second denaturation phase at 95°C, followed by 40 iterative cycles of: Denaturation: 15-second exposure to 95°C. Annealing/Extension: Combined 60-second incubation at 60°C. Final elongation was performed at 72°C for 5 minutes to ensure amplicon integrity. Primers: GPD1L F:5′-CAAATCTTAGCGAGGCTGTGC-3′, R:5′-AAATGAGCTTCAGCCCCTCG-3′ (168 bp), GAPDH F:5′-GGAGCGAGATCCCTCCAAAAT-3′, R:5′-GGCTGTTGTCATACTTCTCATGG-3′ (197 bp), Relative expression calculated via 2−ΔΔCt method.

### 2.4 Pathway enrichment profiling

GSEA v4.2.3 was implemented to identify enriched molecular signatures between GPD1L expression subgroups (high vs low, median cutoff). MSigDB hallmark gene sets were interrogated with 1,000 permutations. Significance thresholds: normalized Enrichment Score (NES) exceeding ±1.6 directional thresholds; Primary Significance: Nominal PP-value < 0.05 derived from Kolmogorov-Smirnov test; Multiple Testing Correction: false discovery rate (FDR) < 0.1.

### 2.5 Cellular model establishment and maintenance

The human colorectal carcinoma cell panel (SW620, HCT116, SW480, DLD-1, LOVO) and non-neoplastic colonic epithelial line NCM460 were procured from the General Surgery Research Laboratory at Tianjin Medical University General Hospital. Four specialized media formulations cultivated cellular systems: McCoy’s 5A, RPMI-1640, Leibovitz L-15, and DMEM, each enriched with 10% fetal bovine serum (FBS) and penicillin-streptomycin(1%) antimicrobial formulation. Standardized *in vitro* conditions were rigorously maintained through incubation in a 5% CO_2_ humidified atmosphere at 37°C, with complete medium replacement executed at 48-hour intervals. To ensure experimental validity, quarterly mycoplasma surveillance was conducted using the One-Step QuickColor Mycoplasma Detection System (Yeasen Biotechnology, Shanghai, Cat#40602ES76) in strict compliance with ISO 17025 testing protocols.

For targeted GPD1L overexpression, 2 μg of the pcDNA3.1-GPD1L mammalian expression plasmid was reconstituted in 100 μL Opti-MEM Medium, while parallel dilutions of Lipofectamine 2000 transfection reagent were prepared in an equivalent volume of Opti-MEM. The lipid-DNA complexes were incubated for 20 minutes at ambient temperature to facilitate nanoparticle formation prior to cellular administration. Transfected cells were collected at 48 hours post-transfection for downstream functional analyses.

### 2.6 Proliferation assay

Proliferative dynamics of GPD1L-overexpressing SW620 and HCT116 cell models were systematically assessed through CCK-8 assay (YEASEN Biotechnology, Cat#40203ES). At 48h post-transfection, cellular suspensions were seeded at a density of 2×10³ cells/well into sterile 96-well microplates (Corning^®^ 3599) through multichannel pipette dispensing, maintaining a standardized volume of 100 μL/well to ensure uniform monolayer formation. Metabolic activity monitoring was conducted at 0, 24, 48, and 72 h intervals through controlled CCK-8 kits incubation: 10 μL additive per well, 37°C incubation for 60 min, followed by spectrophotometric quantification at 450 nm wavelength via TECAN Infinite^®^ M200 microplate reader (Switzerland).

### 2.7 Scratch wound closure analysis

To systematically quantify the impact of GPD1L overexpression on cellular motility, scratch wound assays were conducted using confluent monolayers of SW620 and HCT116 colorectal carcinoma cell models. Genetically modified cellular suspensions (density: 3×10^5^ cells/well) were seeded into 6-well tissue culture-treated plates and incubated for 24 hours post-inoculation to ensure stable adhesion, achieving 90% confluence prior to mechanical wounding with 200-μL sterile pipette tips. Following PBS washes (×2), serum-starved conditions (0% FBS) were maintained during the 48h observation period. Wound closure dynamics were documented at 0/24/48 h intervals under phase-contrast microscopy (Nikon Eclipse Ti2, 100×), with migration rates calculated through ImageJ v1.53 analysis using the formula: Migration rate (%) = [(A0 - At)/A0] ×100, where A represents wound area.

### 2.8 Transwell invasion assay

To establish invasion-permissive substrates, Transwell^®^ polycarbonate filter inserts (Corning^®^ 3422) with defined 8 μm porosity underwent extracellular matrix functionalization using growth factor-reduced Matrigel^®^ (Corning^®^ 354230). The matrix solution was reconstituted through 1:8 volumetric dilution in serum-deprived RPMI-1640 medium, followed by uniform deposition onto membrane surfaces under refrigerated conditions (4°C) for 12-hour polymerization. Cellular suspensions containing 2×10^4^ cells resuspended in 200 μL serum-deprived RPMI-1640 were seeded into apical chambers, while the basolateral chamber received 600 μL chemoattractant medium containing 10% FBS-enriched complete medium. Following standard 24-hour incubation under normoxic conditions (37°C, 5% CO_2_), membranes were subjected to sequential processing: (1) 15-minute fixation with 4% paraformaldehyde (PFA) at ambient temperature; (2) 20-minute nuclear staining with crystal violet (0.1%) solution. Invasion quantification was performed by imaging three randomly selected microscopic fields per membrane using an Olympus IX73 inverted phase-contrast microscopy platform (200× magnification). Digital image analysis for cell enumeration was executed through the Image-Pro Plus 6.0 analytical suite (Media Cybernetics), with transmigration rates calculated relative to control groups.

### 2.9 Western blot analysis

Proteins were extracted from cells using RIPA buffer containing protease inhibitors. After centrifugation (14,000 × rpm, 30 min, 4°C), supernatants were quantified via BCA assay. Samples (20–50 μg protein) were mixed with Laemmli buffer, denatured (95°C, 5 min), and separated on 10% SDS-PAGE gels at 100 V. Proteins were transferred to PVDF membranes (0.45 μm) using a wet transfer system (220mA, 120min). Membranes were blocked with 5% non-fat milk in TBST (1h, RT), then incubated overnight at 4°C with primary antibodies [GPD1L, ab113595, Abcam; HIF-1α, ab179483, Abcam; MMP9, ab228402, Abcam; β-actin, ab8227, Abcam] diluted in blocking buffer. After TBST washes, membranes were probed with HRP-conjugated secondary antibodies (1h, RT). Signals were visualized by ECL and analyzed using ImageJ.

### 2.10 Statistical analysis

Biostatistical analyses were executed via R version 3.5.2 (R Foundation) and GraphPad Prism 8.0.1 (GraphPad Software). To assess non-parametric associations between GPD1L expression and clinicopathological parameters, Wilcoxon signed-rank tests and Kruskal-Wallis tests were used. Survival trajectories were generated through Kaplan-Meier estimation with between-group comparisons ascertained by log-rank testing. Multivariable survival analysis was conducted through Cox proportional hazards regression utilizing the R statistical platform (version 4.3.1) with survival package. Continuous variables were analyzed using parametric methods: independent two-group comparisons employed Student’s t-test, while multi-group comparisons implemented one-way ANOVA. For non-normally distributed variables, non-parametric alternatives were applied: Mann-Whitney U test and Kruskal-Wallis H-test. Categorical variable associations were interrogated through Pearson’s chi-square contingency testing with Yates’ continuity correction where appropriate. All inferential analyses adopted a bidirectional α threshold of 0.05 for significance determination.

## 3 Results

### 3.1 GPD1L expression alterations in colorectal tissues

**Figure 1:**
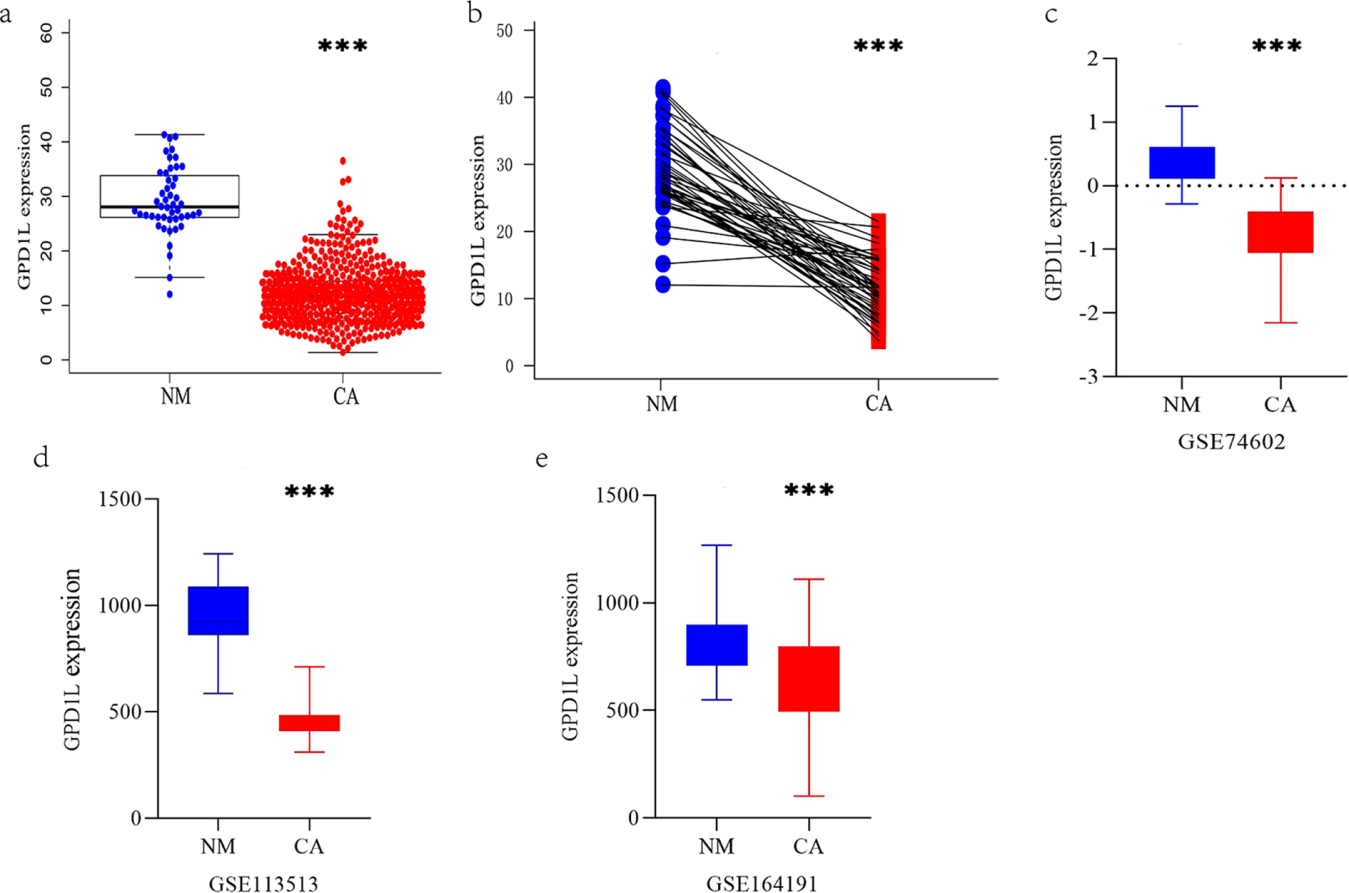
The expression differences of GPD1L in multiple datasets were analyzed. **(a)** Differential GPD1L expression profiling between malignant colorectal tissues (n=554) and non-cancerous controls (n=48), **(b)** Expression difference of GPD1L in 47 CRC tissues and donor-matched normal tissues (TCGA database), **(c)** Expression difference of GPD1L in 30 CRC tissues and donor-matched normal tissues (GSE74602), **(d)** Differences in the expression of GPD1L in 14 CRC tissues and donor-matched normal tissues (GSE113513), **(e)** Differences in the expression of GPD1L in whole blood genes between 62 healthy controls and 59 CRC patients (GSE164191), (***P<0.001).

**Figure 2:**
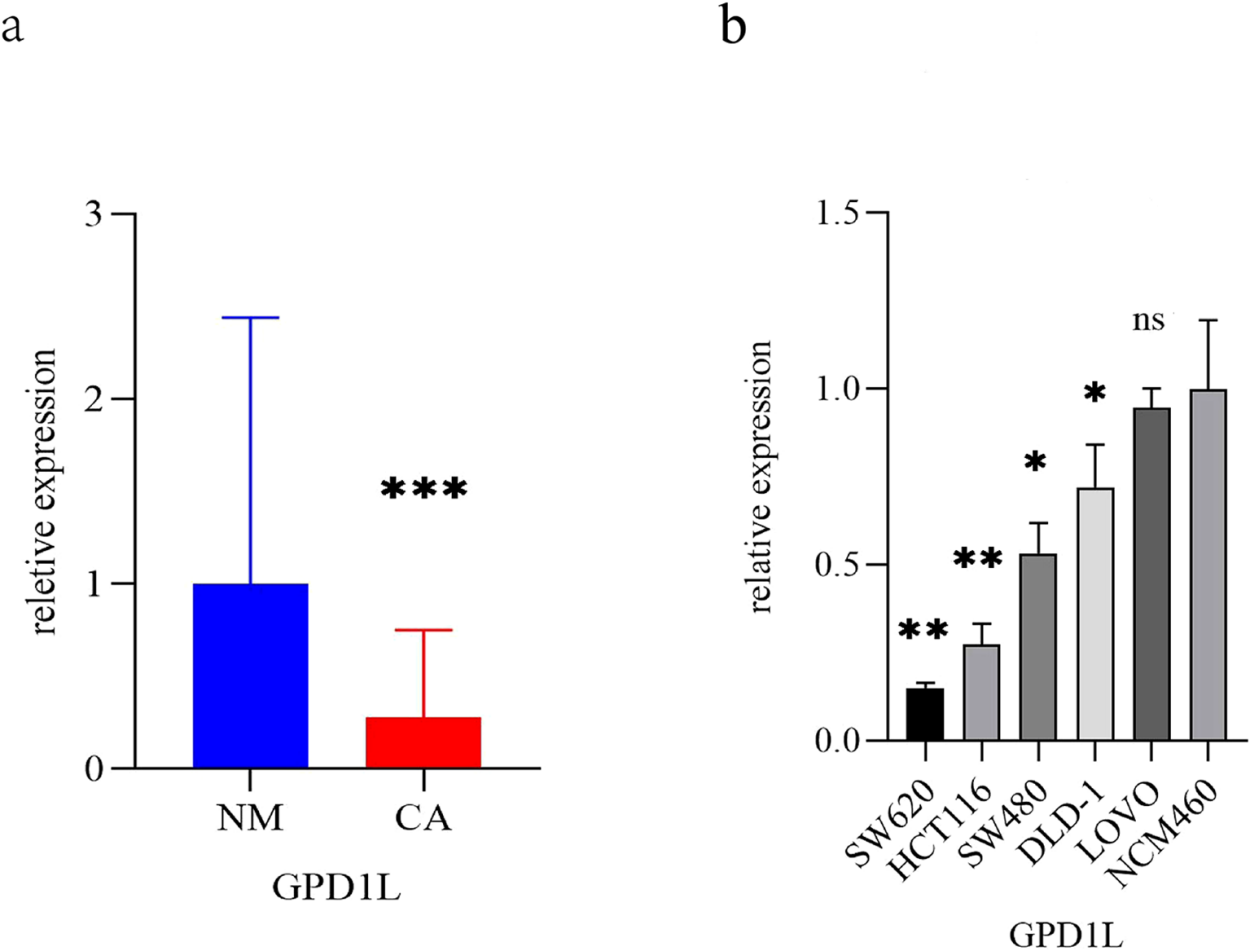
The expression differences of GPD1L in clinical specimens and CRC cells. **(a)** Differences in the expression of GPD1L in 58 CRC patients and donor-matched normal tissues (***P<0.001), **(b)** Differences in the expression of GPD1L in normal colonic epithelial cell lines (NCM460) and five CRC cell lines (SW620, HCT1161, SCT116, DLD 480 and LOVO) (ns P>0.05, *P<0.05, **P<0.01, ***P<0.001).

TCGA cohort analysis demonstrated significantly reduced GPD1L expression in 554 CRC tissues with 48 normal controls (P<0.001, **Figure 1a**). Paired-sample analysis of 47 CRC cases confirmed tumor-specific downregulation (P<0.001, **Figure 1b**). External validation using GEO datasets revealed consistent patterns: GSE74602 (30 tumor-normal pairs, P<0.001, **Figure 1c**) and GSE113513 (14 pairs, P<0.001, **Figure 1d**) both exhibited decreased GPD1L in malignant tissues. Systemic analysis of GSE164191 further identified reduced GPD1L levels in whole blood samples from 59 CRC patients versus 62 healthy controls (P<0.001, **Figure 1e**). Our institutional cohort (58 paired specimens) replicated these findings, showing diminished GPD1L expression in tumors (P<0.001, **Figure 2a**). Cellular-level analysis disclosed significantly higher GPD1L levels in non-malignant NCM460 cells compared to CRC lines (HCT116/DLD-1/SW480: P<0.01; SW620: P<0.05, **Figure 2b**).

### 3.2 GPD1L downregulation correlates with disease progression

**Figure 3:**
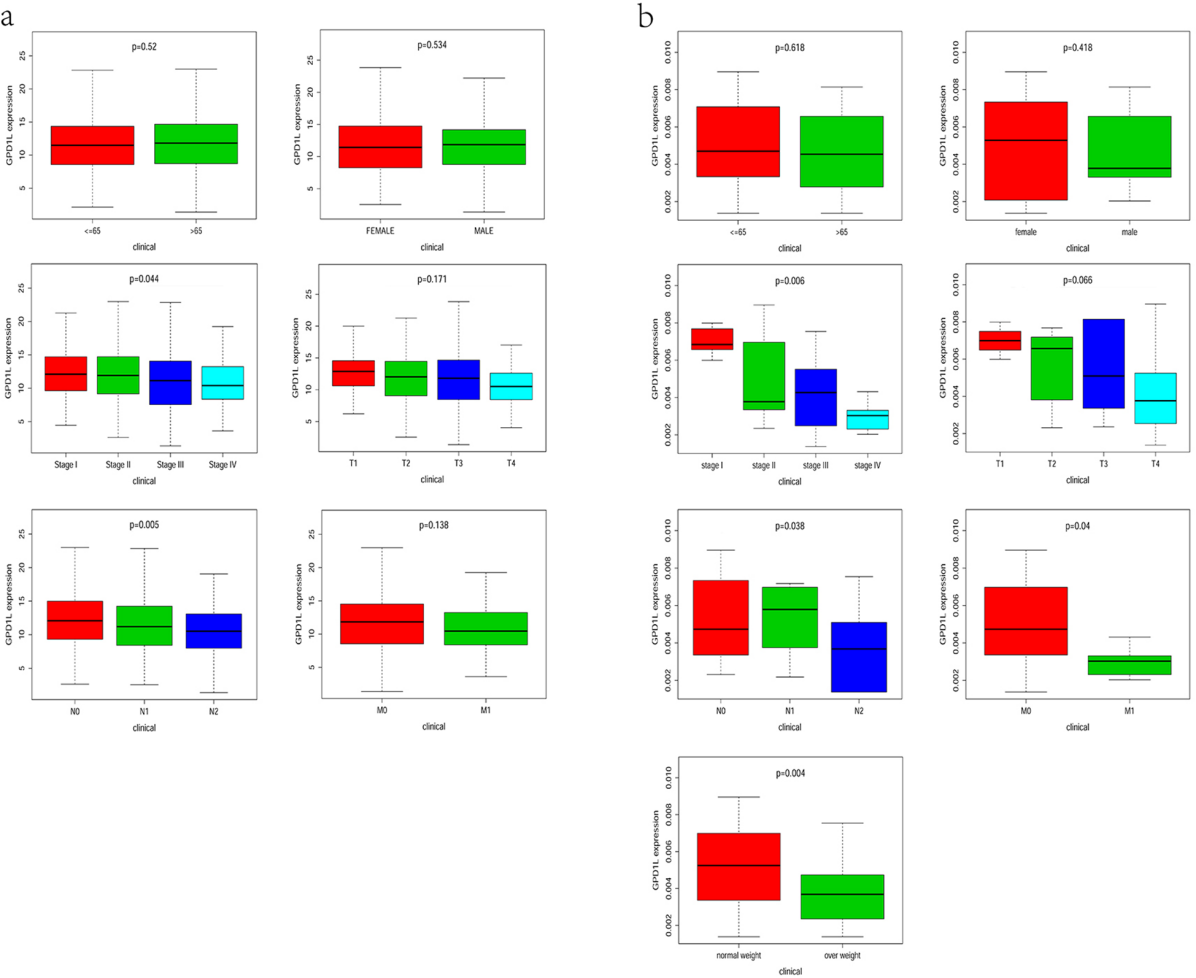
Relationship between GPD1L expression level and clinicopathological features. **(a)** TCGA database, **(b)** Data from the center.

Stratification analysis of 533 TCGA-CRC cases revealed significant GPD1L expression variations across nodal involvement (N-stage: P=0.005) and overall clinical staging (P=0.044) (**Figure 3a**). No associations were observed with age, sex, primary tumor invasive depth (T-stage), or distant metastasis (M-stage). Complementing these findings, our institutional cohort analysis (n=58) demonstrated progressive GPD1L downregulation correlating with metastatic dissemination (M-stage: P=0.04), advanced nodal metastasis (N-stage: P=0.038) and advanced clinical staging (P=0.006). Notably, elevated body mass index (>25 kg/m²) showed inverse correlation with GPD1L levels (P=0.004) (**Figure 3b**).

### 3.3 Prognostic implications of GPD1L downregulation

**Figure 4:**
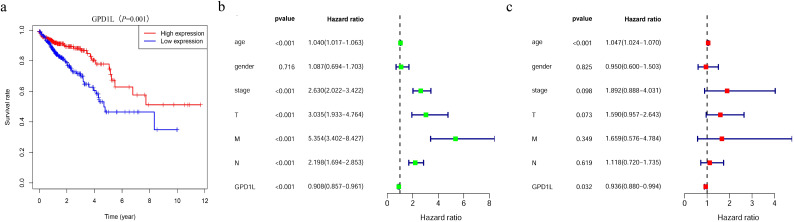
Patients with CRC with low GPD1L expression have a worse prognosis. **(a)** The relationship between GPD1L expression and overall survival in CRC patients in the TCGA database, **(b)** Univariate Cox regression analysis of GPD1L expression and clinicopathological features, **(c)** Multivariate Cox regression analysis of GPD1L expression and clinicopathological features.

Survival trajectory analysis via the Kaplan-Meier method demonstrated a pronounced decline in overall survival rates among CRC cohorts exhibiting attenuated GPD1L expression (P=0.001, **Figure 4a**). Univariate Cox regression identified GPD1L downregulation (HR=0.908, 95%CI:0.857-0.961; P<0.001) as a prominent prognostic determinant alongside established clinical parameters, including advanced T-stage (HR=3.035, 95%CI:1.933-4.764), nodal metastasis (HR=2.198, 95%CI:1.694-2.853), distant dissemination (HR=5.354, 95%CI:3.402-8.427), age (HR=1.040, 95%CI:1.017-1.063), and advanced clinical staging (HR=2.630, 95%CI:2.023-3.422), all demonstrating P<0.001 significance (**Figure 4b**). Following comprehensive adjustment for potential clinical confounders including tumor stage, differentiation status, and so on, multivariable Cox proportional hazards regression analysis identified GPD1L transcriptional levels (adjusted HR=0.936, 95% CI: 0.880-0.994; P=0.032) and patient age (adjusted HR=1.047, 95% CI: 1.024-1.070; P<0.001) as statistically independent prognostic determinants of overall survival (OS). These findings persisted after rigorous sensitivity analysis, confirming the robustness of both biomarkers in predicting long-term oncological outcomes (**Figure 4c**).

### 3.4 GSEA enrichment analysis

**Figure 5:**
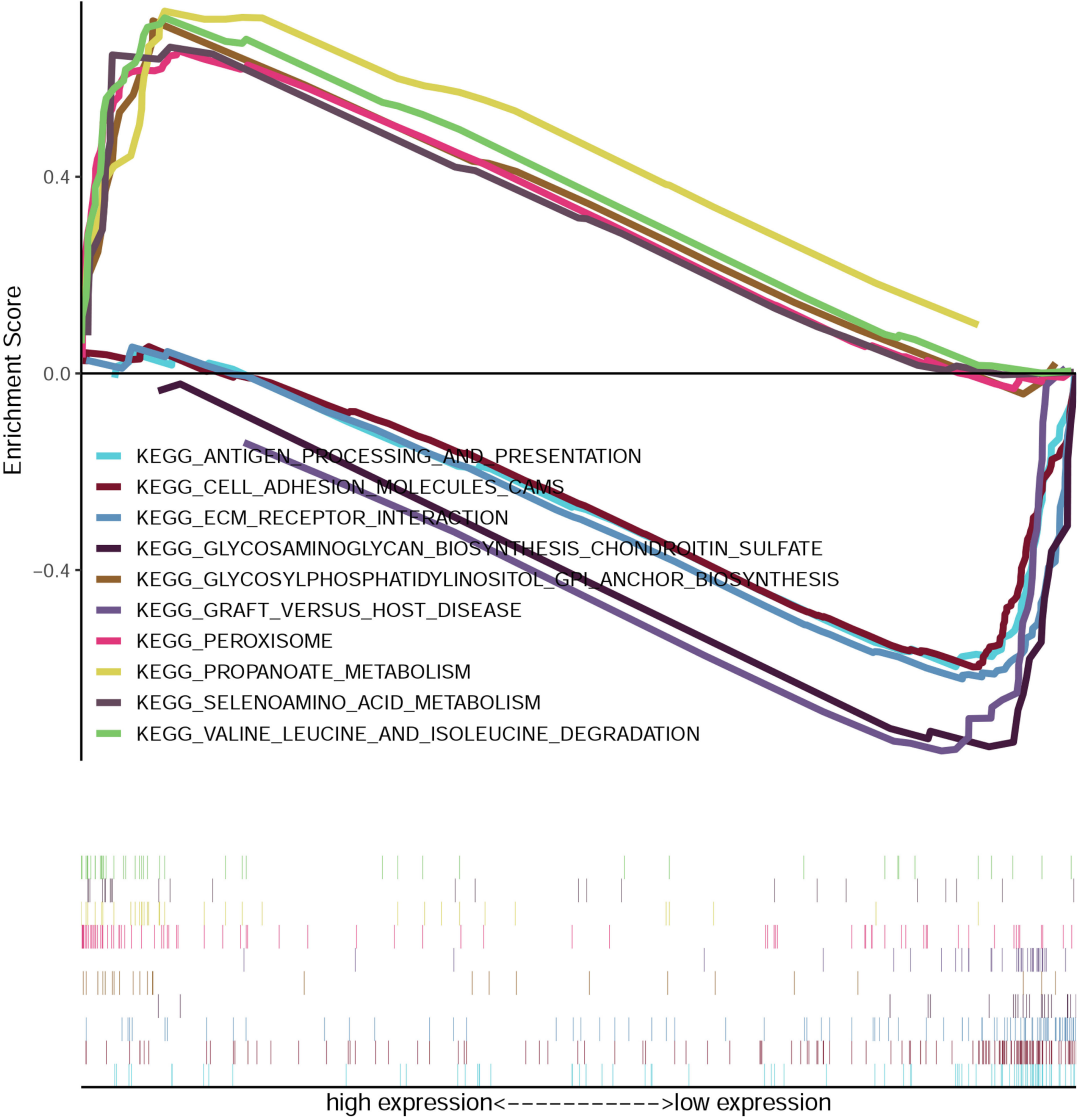
Enrichment plots from the gene set enrichment analysis (GSEA). GSEA results showing differential enrichment of genes in KEGG with GPD1L expression.

Gene Set Enrichment Analysis (GSEA) of CRC samples stratified by GPD1L expression levels revealed distinct functional signatures. Functional annotation of KEGG pathways revealed statistically significant enrichment (Benjamini-Hochberg adjusted FDR<0.05) within the GPD1L-low expression cohort, with predominant activation of adaptive immune response mechanisms, particularly antigen processing and presentation pathways, cell adhesion molecules (CAMs) and extracellular matrix (ECM) receptor interactions. Conversely, GPD1L-high specimens exhibited predominant activation of metabolic pathways, notably glycosylphosphatidylinositol-mediated membrane anchoring systems, catabolic processing of branched-chain amino acids, and peroxisome-related metabolism (**Figure 5**).

### 3.5 GPD1L overexpression inhibits cellular proliferation

**Figure 6:**
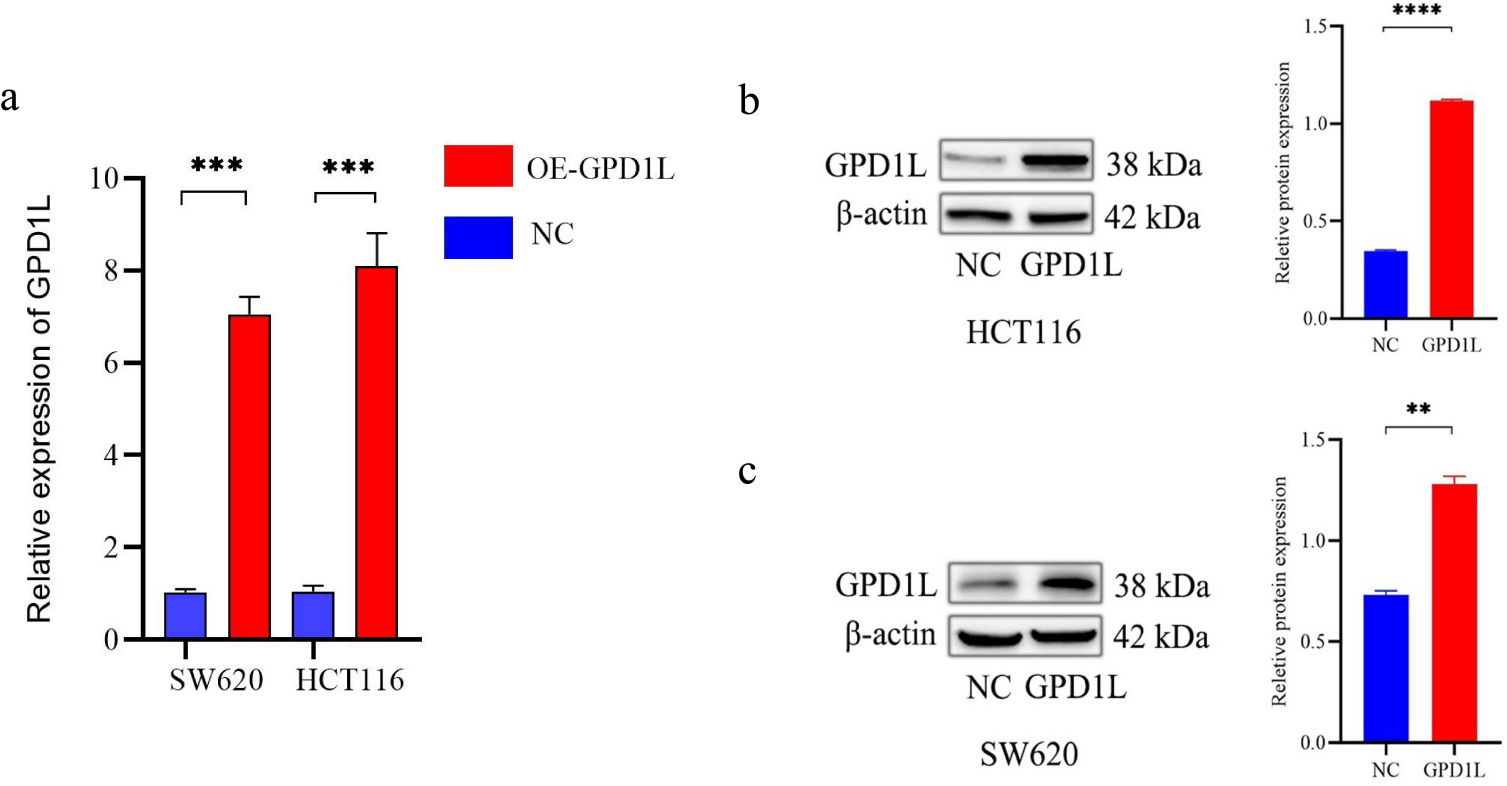
Transfected with GPD1L overexpression plasmid. **(a)** Transfection efficiency at the mRNA level of GPD1L overexpression plasmid, **(b)** Transfection efficiency at the protein level of GPD1L overexpression plasmid in HCT116 cells, **(c)** Transfection efficiency at the protein level of GPD1L overexpression plasmid in SW620 cells (**P<0.01, ***P<0.001, ****P<0.0001).

**Figure 7:**
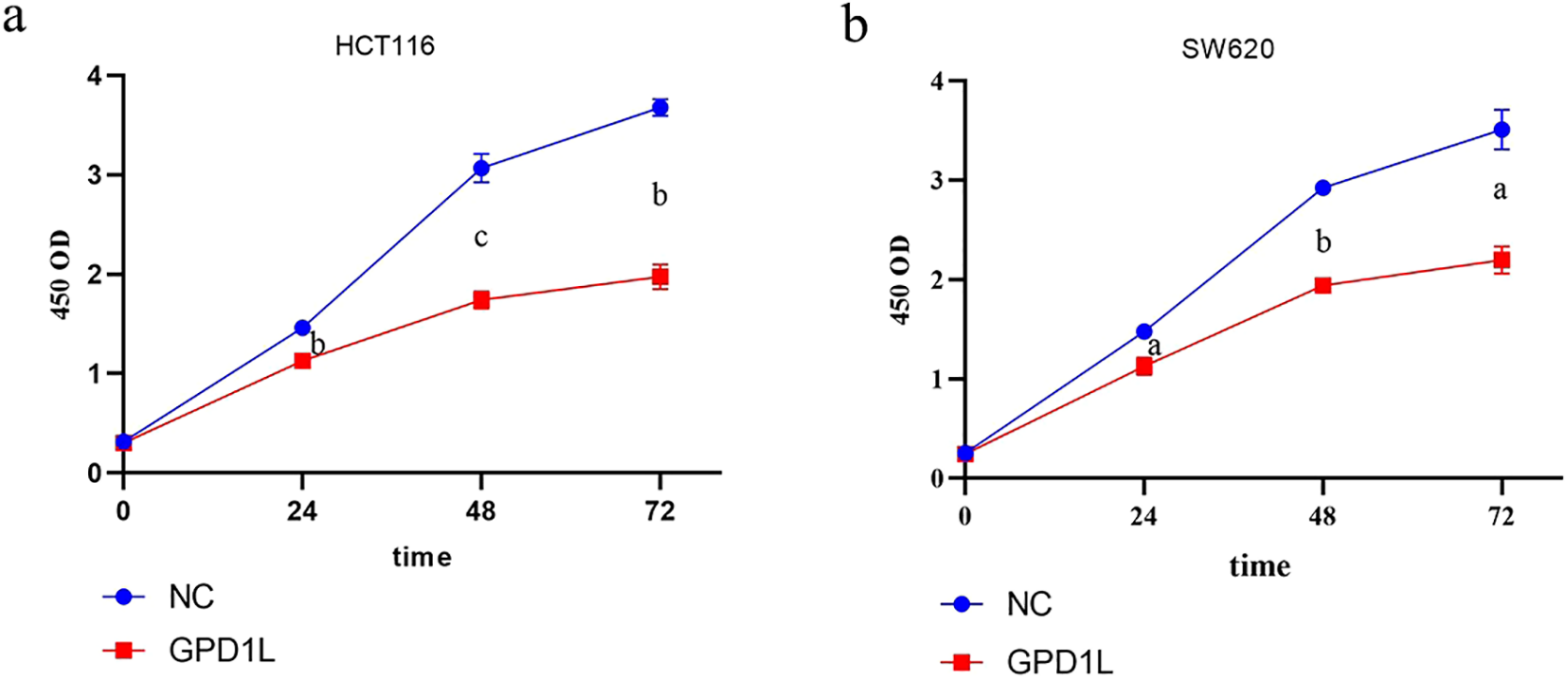
Results of proliferation capacity analysis. **(a)** Changes in the proliferation capacity of HCT116 after GPD1L overexpression, **(b)** Changes in the proliferation capacity of SW620 (^a^P<0.05, ^b^P<0.01, ^c^P< 0.001).

Quantitative PCR analysis confirmed differential GPD1L expression across colorectal cancer cell (SW480, SW620, DLD-1, HCT116, LOVO) and normal colonic NCM460 cells. Significantly lower GPD1L levels were observed in SW480 (P<0.05), SW620 (P<0.05), DLD-1 (P<0.05) and HCT116 (P<0.01), compared to NCM460 controls (**Figure 2b**). Given the minimal expression in HCT116 and SW620, these lines were selected for functional studies. Transfection with GPD1L overexpression plasmid (OE) significantly elevated mRNA and protein levels in both SW620 and HCT116 (P<0.001) versus empty vector controls (**Figure 6**). CCK-8 assays demonstrated marked proliferation inhibition in OE groups: HCT116 and SW620 proliferation decreased compared to baseline controls(P<0.001) (**Figure 7**).

### 3.6 GPD1L reconstitution suppresses metastatic potential

**Figure 8:**
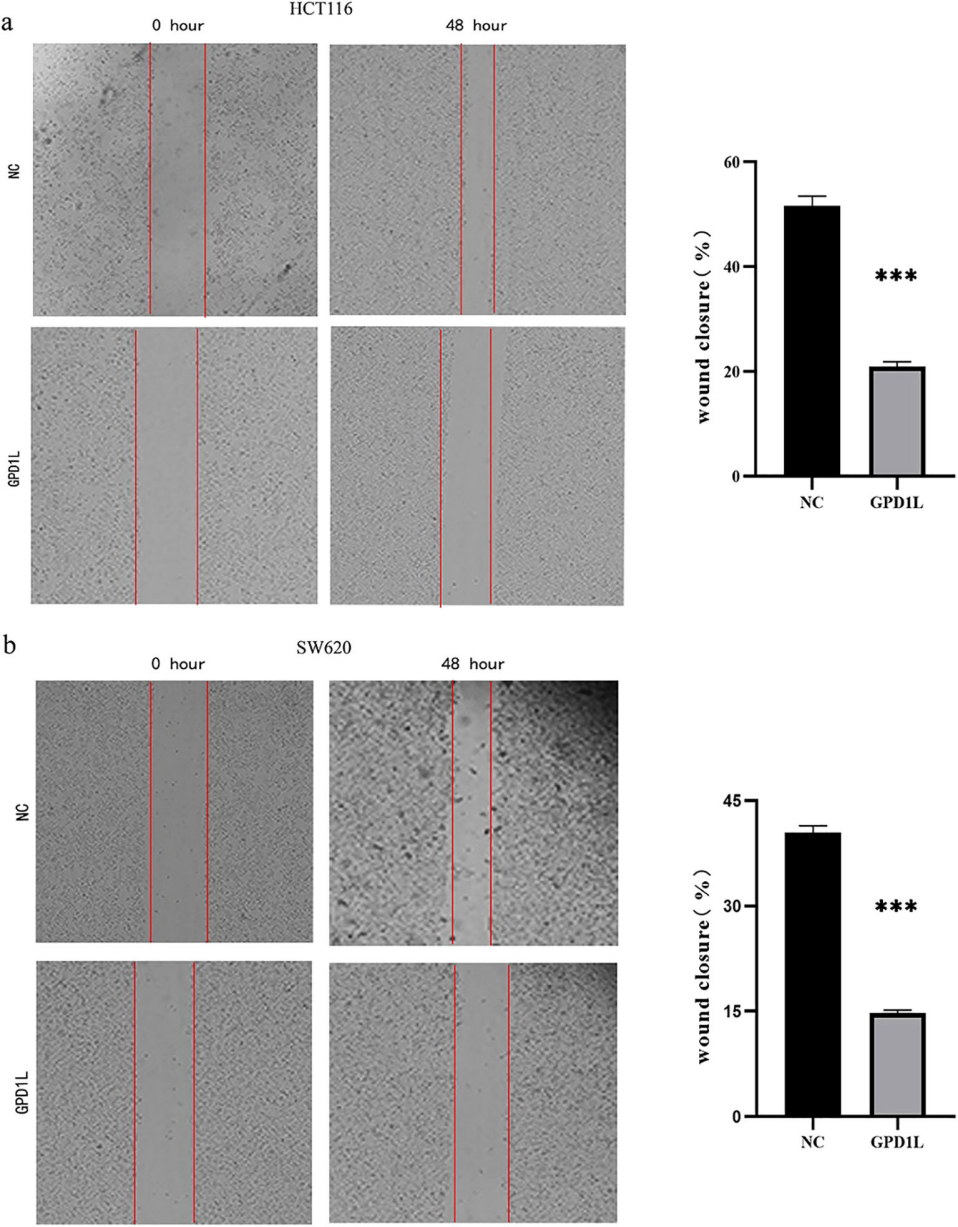
Results of scratch wound closure analysis. **(a)** HCT116 migration ability decreased after GPD1L overexpression, **(b)** SW620 migration ability decreased after GPD1L overexpression (***P<0.001).

**Figure 9:**
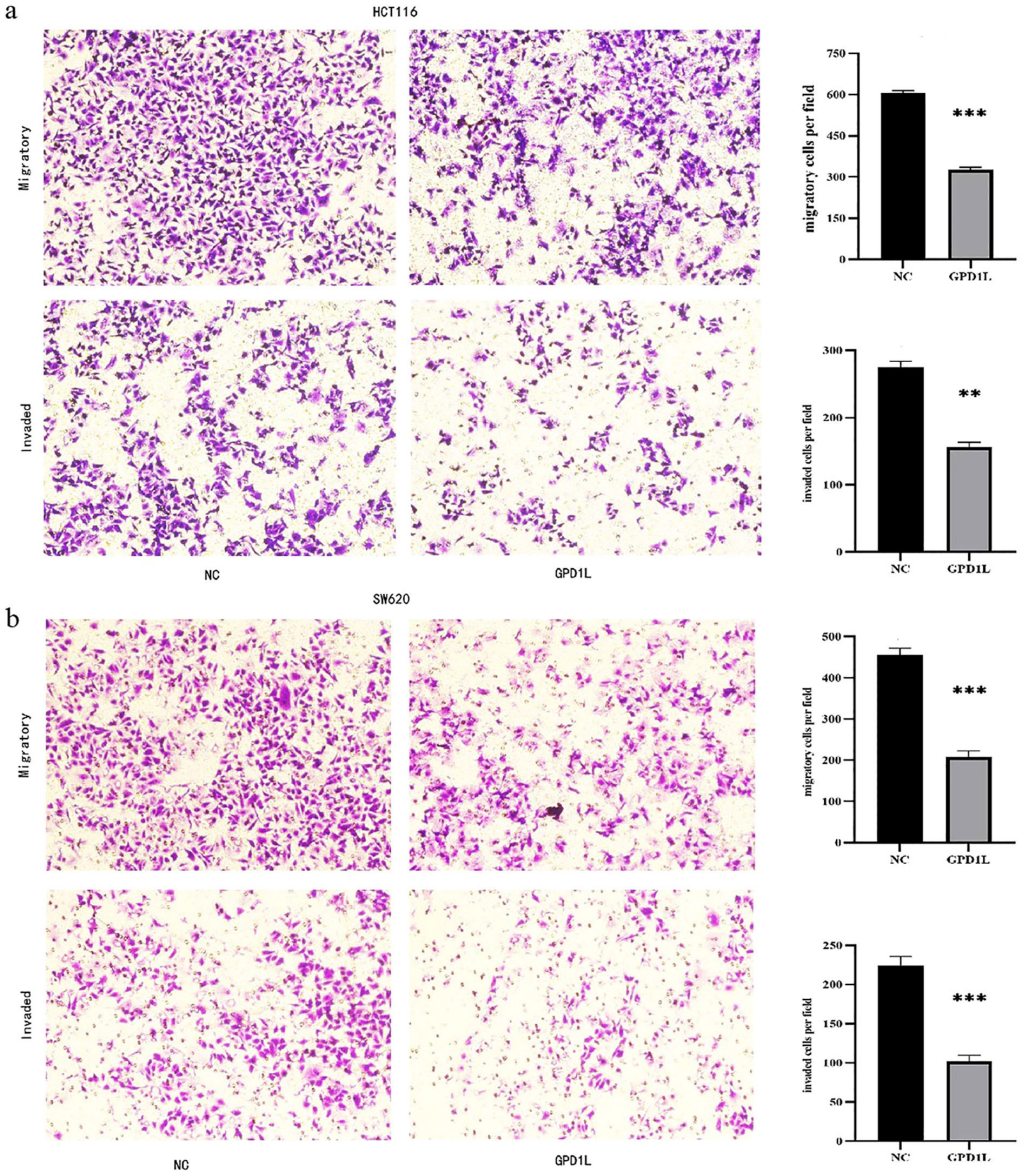
Transwell experimental results. **(a)** The migration and invasion ability of HCT116 decreased after overexpression of GPD1L, **(b)** The migration and invasion ability of SW620 decreased after overexpression of GPD1L (**P<0.01, ***P<0.001).

Scratch wound closure analysis demonstrated significant attenuation of migratory capacity in GPD1L-overexpressing cells: HCT116 migration decreased from 51.63 ± 1.84% to 14.77 ± 0.40% (P<0.001), and SW620 from 40.43 ± 0.97% to 20.90 ± 0.96% (P<0.001) versus controls (**Figure 8**). Transwell validation confirmed this inhibitory effect, revealing reduced migratory cell counts in HCT116 (605.0 ± 9.2 vs 326.7 ± 8.50 cells/field, P<0.001) and SW620 (455.3 ± 17.2 vs 208.0 ± 14.0 cells/field, P<0.001). Invasion capacity was similarly impaired, with HCT116 invaded cells decreasing from 274.3 ± 9.6 to 155.7 ± 7.5 (P<0.01), and SW620 from 224.7 ± 10.9 to 101.7 ± 8.5 (P<0.001) (**Figure 9**).

### 3.7 GPD1L potentially affects the biological behavior of CRC cells through altered HIF-1α stabilization and MMP9 transcriptional activity

**Figure 10:**
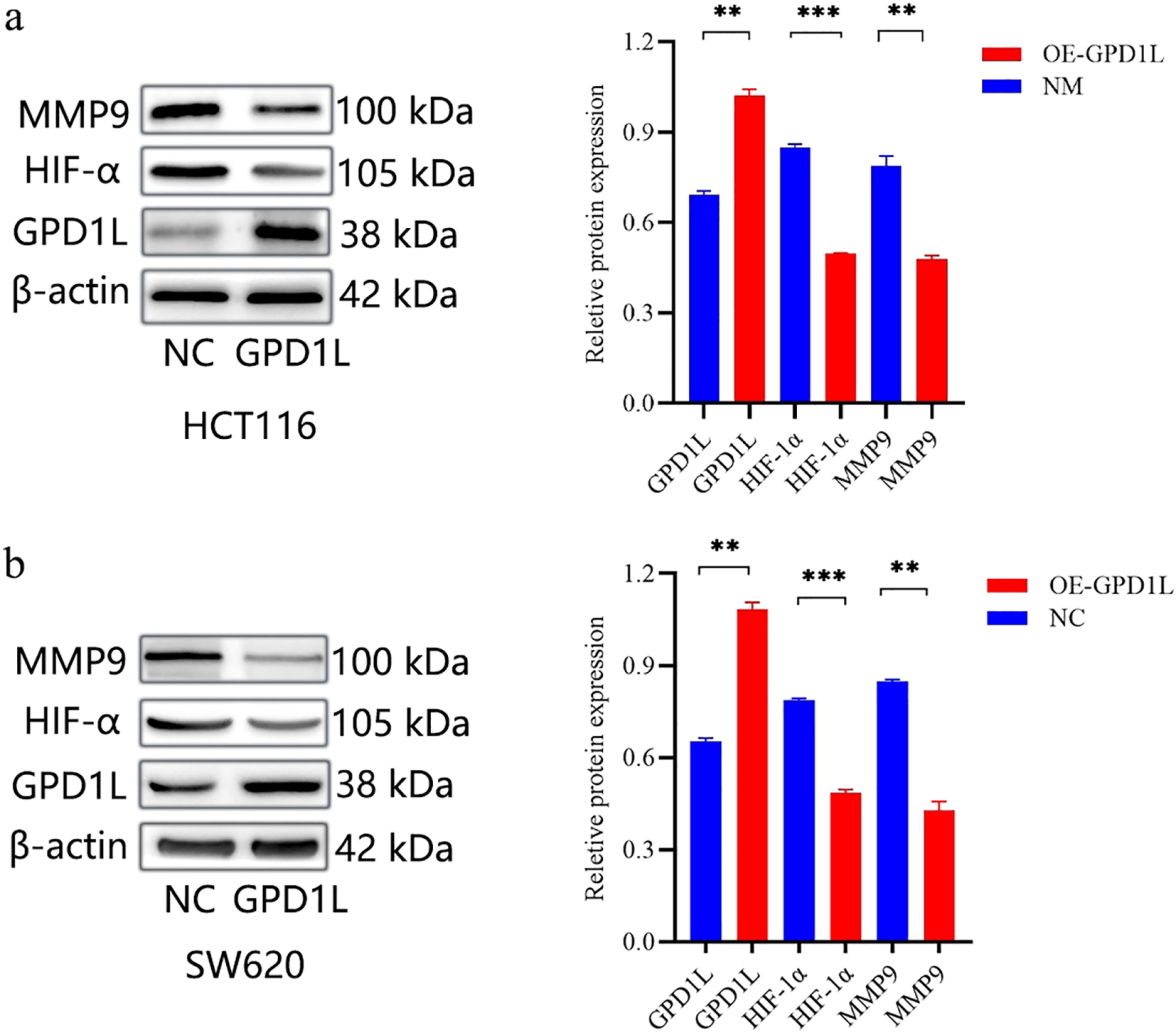
Genetic modulation of GPD1L overexpression altered HIF-1α stabilization and MMP9 transcriptional activity in CRC cell lines. **(a)** Effect of GPD1L overexpression on the expression levels of HIF-1α and MMP9 in HCT116 cells, **(b)** Effect of GPD1L overexpression on the expression levels of HIF-1α and MMP9 in SW620 cells (**P<0.01, ***P<0.001).

We used GSEA to analyze the potential mechanism by which GPD1L affects CRC cells. The results are shown in **Figure 5**. Low expression of GPD1L activated the cell adhesion molecules (CAMs) and extracellular matrix (ECM) receptor interactions molecular pathways, and MMP9 is closely related to these two signaling pathways (12). Many studies have shown that GPD1L may affect the activity of HIF-1α. Therefore, we speculate that GPD1L may affect the biological behavior of CRC cells by affecting the expression of these two molecules. Proteins of CRC cells overexpressing GPD1L were extracted and WB analysis were performed. The results showed that when GPD1L was overexpressed, the expression of HIF-1α and MMP9 in HCT116 cells was reduced, and the same results were shown in SW620 cells (**Figure 10**). These results supported our hypothesis that GPD1L can affect the malignant behavior of CRC cells by affecting the activity of HIF-1α and the expression of MMP9.

## 4 Discussion

The global surge in obesity prevalence, attributable to calorically dense dietary patterns and reduced physical activity, now represents a pivotal challenge in preventive oncology. Epidemiological studies consistently implicate adiposity as an independent risk modulator for colorectal carcinogenesis, demonstrated significant epidemiological correlations with dysmetabolic conditions including cardiovascular pathologies, type II diabetes mellitus and multiple malignancies (7, 13, 14). Colorectal cancer (CRC), in particular, exhibits a well-documented epidemiological link to obesity, with meta-analyses revealing a 1.3-fold increased risk in obese individuals (BMI >30 kg/m²) (15). Despite this clinical association, the molecular mechanisms bridging adiposity and colorectal carcinogenesis remain poorly elucidated. Our study addresses this gap by identifying GPD1L, a gene recently implicated in obesity pathogenesis through genome-wide association studies (16), as a novel tumor suppressor in CRC, thereby providing mechanistic insights into obesity-driven oncogenesis.

Located at chromosomal locus 3p22.3, the GPD1L gene encodes a dehydrogenase enzyme critical to glycerol phosphate metabolism. First identified through the NIH Mammalian Gene Collection initiative in 2002, this gene initially gained recognition for its regulatory functions in cardiac sodium channel dynamic (17), GPD1L was first characterized for its role in cardiac sodium channel trafficking and association with Brugada syndrome (8, 10). Subsequent investigations have expanded its functional repertoire, revealing its capacity to destabilize hypoxia-inducible factor 1-alpha (HIF-1α) through prolyl hydroxylase activation (18), thereby modulating angiogenesis via VEGF regulation (19). Intriguingly, recent studies highlight GPD1L’s tumor-suppressive potential across diverse malignancies, including HER2-negative breast cancer (20), esophageal squamous cell carcinoma (21), and gastric carcinoma (22), though its role in CRC remained unexplored prior to this investigation.

Our multi-platform analysis of TCGA and GEO datasets revealed consistent GPD1L downregulation in CRC tissues compared to normal mucosa (**Figure 1**), a finding validated in our institutional cohort (n=58 tumor-normal pairs; **Figure 2a**). Clinically, diminished GPD1L expression correlated with advanced nodal metastasis (N-stage), distant dissemination (M-stage), and poorer survival outcomes (**Figures 3a**, **4a**), establishing its prognostic relevance. Multivariate Cox regression confirmed GPD1L as an independent prognostic indicator (HR=0.936, P=0.032; **Figure 4c**), reinforcing findings from gastric cancer studies where GPD1L loss predicted aggressive phenotypes (22). Notably, overweight patients exhibited accentuated GPD1L downregulation (**Figure 3b**), aligning with preclinical models demonstrating diet-induced obesity reduces hepatic GPD1L expression by 63% (P<0.001) (11), suggesting a potential mechanism for obesity-associated CRC progression.

Functional validation in low-expressing CRC models (HCT116/SW620) demonstrated GPD1L’s multimodal tumor suppression: Proliferation inhibition (P<0.001; **Figure 7**), consistent with its HIF-1α regulatory role (23) and anti-angiogenic effects via VEGF suppression (24); Metastatic constraint, evidenced by 62–68% migration attenuation (**Figures 8**, **9**), mirroring observations in head-neck squamous carcinoma where GPD1L inversely correlated with recurrence (25); Metabolic reprogramming revealed through GSEA, showing GPD1L-high tumors enriched in branched-chain amino acid degradation (**Figure 5**), a pathway linked to tumor suppression in gastric cancer (22).

Mechanistically, the dichotomous pathway activation—immune/ECM remodeling in GPD1L-low versus metabolic regulation in GPD1L-high tumors (**Figure 5**)—suggests GPD1L modulates tumor-stroma crosstalk. This aligns with its recently identified role in miR-210-mediated HIF-1α regulation under hypoxia (26), potentially explaining the metabolic plasticity observed in CRC progression. Our results confirmed this mechanism. When GPD1L was overexpressed in CRC cells, we found that the expression of HIF-1α and MMP9 was reduced (**Figure 10**).

While our integrated approach (bioinformatics, clinical cohorts, functional assays and molecular mechanism) provides robust evidence, limitations include the retrospective clinical analysis and lack of *in vivo* validation. Future studies should explore isoform-specific effects, given GPD1L’s known splice variants in cardiovascular systems, and therapeutic potential in obesity-associated CRC models.

## 5 Conclusion

This study establishes GPD1L downregulation as a molecular hallmark of CRC progression, mechanistically linking obesity-associated metabolic dysregulation to metastatic dissemination. The conserved tumor-suppressive activity across epithelial malignancies positions GPD1L as a promising therapeutic target for metabolic syndrome-associated cancers.

## Data Availability

The datasets presented in this study can be found in online repositories. The names of the repository/repositories and accession number(s) can be found below: https://www.ncbi.nlm.nih.gov/.
